# Surgical Treatment of Osteopetrosis-Related Femoral Fractures: Two Case Reports and Literature Review

**DOI:** 10.1155/2014/891963

**Published:** 2014-01-21

**Authors:** Ahmet Aslan, Yakup Barbaros Baykal, Emin Uysal, Tolga Atay, Vecihi Kirdemir, Metin Lütfi Baydar, Nevres Hürriyet Aydoğan

**Affiliations:** ^1^Departments of Orthopaedics and Traumatology, Afyonkarahisar State Hospital, Orhangazi Mh. Nedim Helvacıoglu Cd. No. 73 Uydukent, 03100 Afyonkarahisar, Turkey; ^2^Departments of Orthopaedics and Traumatology, Medical Faculty, Süleyman Demirel University, Isparta, Turkey; ^3^Department of Emergency and First Aid, Bağcılar Education and Research Hospital, Istanbul, Turkey; ^4^TBMM, Ankara, Turkey; ^5^Departments of Orthopaedics and Traumatology, Ankara Education and Research Hospital, Ankara, Turkey

## Abstract

Osteopetrosis is a rare hereditary disease which is characterized by increased bone density. Bone resorption is insufficient or fails due to the osteoclast defect in osteopetrosis. Half of the patients are asymptomatic and diagnosed incidentally or based on the presence of fracture. Adult onset osteopetrosis usually presents with hip and proximal femoral fractures. Internal fixation can be performed; however, technical challenges may be experienced due to increased bone density. As in other fractures, nonunion or varus malunion of these fractures may occur. Although rare, osteopetrosis may complicate treatment of fractures in such patients. In this study, we aimed to present two new cases of ADO type II with an osteopetrotic femoral fracture along with the clinical and radiological findings in the light of a comprehensive literature review. Orthopaedics surgeons should be aware of intraoperative technical difficulties and possible postoperative complications during the follow-up period. Investigation would be beneficial for the diagnosis of osteopetrosis such the patient with fractures who has minor trauma history and increased bone density in radiography.

## 1. Introduction

Osteopetrosis is a rare hereditary disease which is characterized by increased bone density [1–3]. Bone resorption is insufficient or fails due to the osteoclast defect in osteopetrosis. The disease usually presents with increased bone mass and generalized osteosclerosis [1, 4–9]. It has three clinical forms based on the age of onset, inheritance pattern, and clinical features: (i) infantile or malignant osteopetrosis, (ii) intermediate, and (iii) adult onset or benign osteopetrosis [1, 4, 5, 10–12]. Adult benign autosomal dominant osteopetrosis has two distinct phenotypic variants [1, 6–8]. Osteopetrosis tarda, which is also known as marble bone disease, is a subtype of autosomal dominant osteopetrosis type II (ADO type II) [1, 4, 5, 9]. It is characterized by clinically minor trauma-related fractures and typical radiographic findings of failure of tubulation and a “bone within a bone” appearance. Half of the patients are asymptomatic and diagnosed incidentally or based on the presence of fracture. Adult onset osteopetrosis usually presents with hip and proximal femoral fractures [3, 10]. Internal fixation can be performed; however, technical challenges may be experienced due to increased bone density. As in other fractures, nonunion or varus malunion of these fractures may occur. Although rare, osteopetrosis may complicate treatment of fractures in such patients. There are usually as case reports regarding the treatment of osteopetrosis-related fractures in the literature [9].

In this study, we aimed to present two new cases of ADO type II with an osteopetrotic femoral fracture along with the clinical and radiological findings in the light of a comprehensive literature review.

Informed consents were obtained from the patients and the study protocol was approved by the local ethics committee.

## 2. Case Presentation

**Figure 1:**
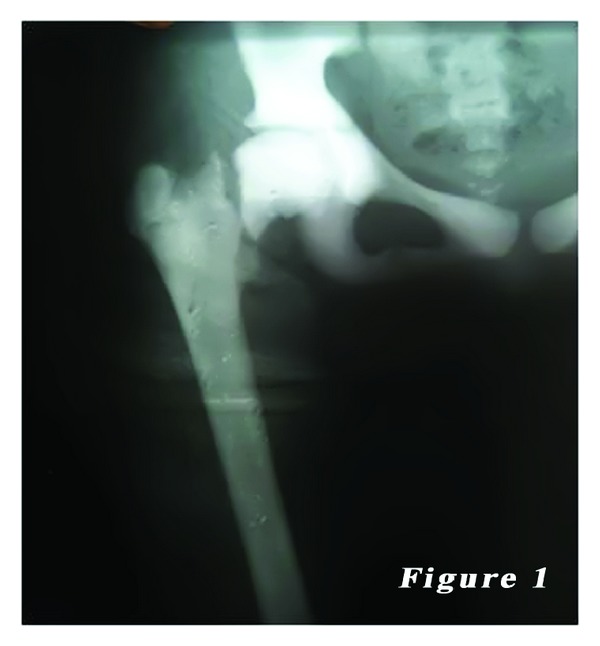
14-year-old female patient (first case) preoperative radiography of the right femoral neck fracture.

**Figure 2:**
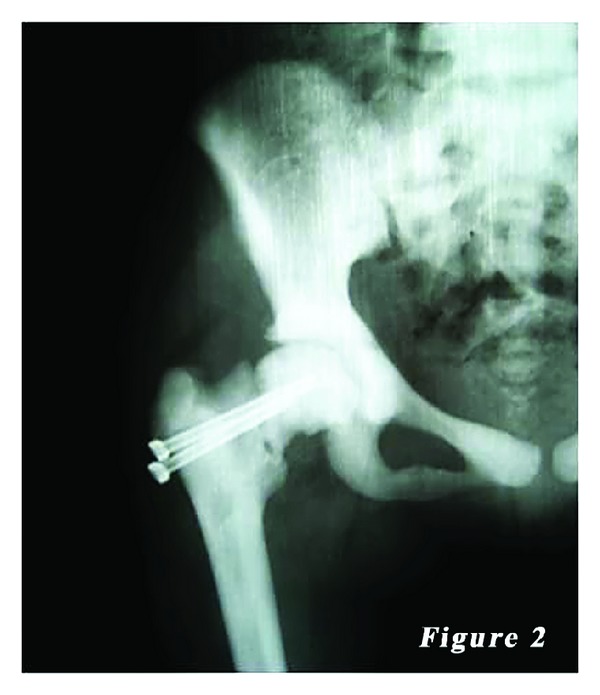
Postoperative radiography of the first patient.

**Figure 3:**
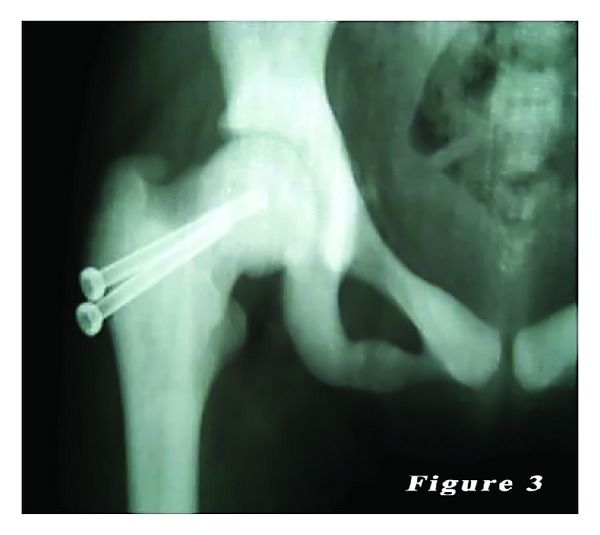
Her last radiography. It looks like fracture union.

*Case 1.* A 14-year-old female patient was admitted to our emergency department with the complaints of hip pain and the inability to walk, which occurred after a minor fall, based on the patient history. There was no previous fracture in her medical history. However, family history of the patient showed a similar bone disease. Physical examination revealed pain with palpation as well as loss of hip range of motion. Ocular and neurological examination demonstrated bilateral loss of vision. Other systemic examinations showed normal vital findings. Laboratory test results were within normal limits, except mild anemia. Plain X-ray showed a mildly displaced fracture of the right femoral neck with a dense sclerotic line. Based on the patient history, physical examination findings, and laboratory and imaging test results, along with consultation remarks, the patient was diagnosed with osteopetrosis tarda and osteopetrotic femoral proximal fracture. Following closed reduction, a lateral straight mini-incision was performed under fluoroscopic visualization. Drilling was highly difficult and time-consuming, and one of the drill tips was bent. However, osteosynthesis was performed using two 4.5 mm spongious screws without any other complications. Fluoroscopic assessment revealed an adequate reduction and fixation. Based on the intraoperative examination, the range of motion of the hip was within normal limits. Bone specimens which were obtained by drilling and the intraoperative biopsy were sent for pathological examination. No perioperative complications were observed. In the postoperative period, there was no scar-related complication, either. The patient was referred to the rehabilitation program on the third postoperative day and sutures were removed at 12 days. She was mobilized without loading on the healing fracture, using a crutch at two weeks and with partial loading at six weeks. The histopathological examination confirmed the diagnosis of osteopetrosis. No complications including infection, nonunion, or avascular necrosis (AVN) were observed during the 12-month follow-up period. The patient was able to walk without pain and using any assistance. Plain radiographs are shown in Figures 1, 2, and 3.

**Figure 4:**
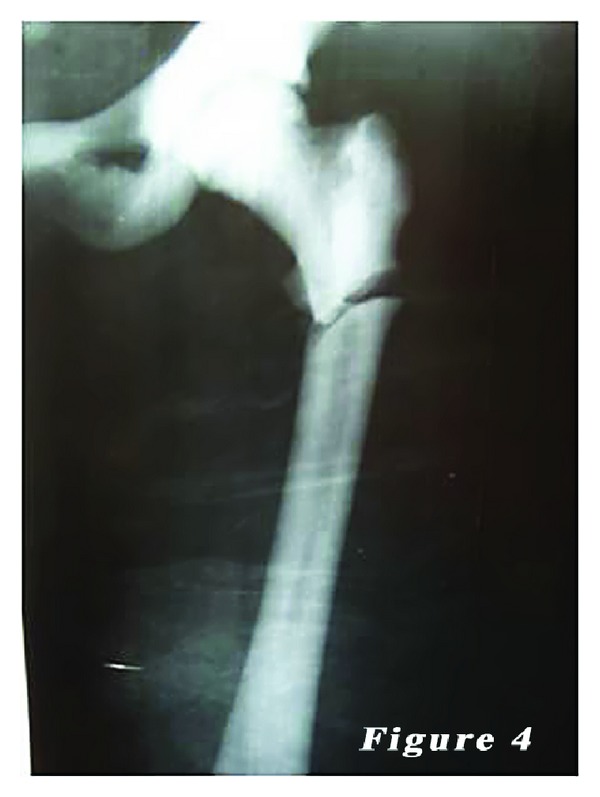
24-year-old female patient (second case) preoperative radiograph of the left femur subtrochanteric fractures.

**Figure 5:**
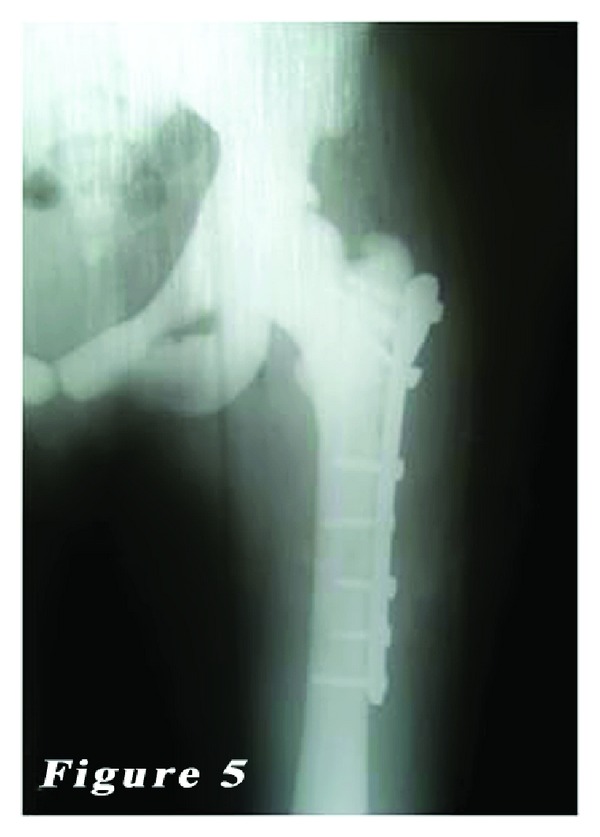
Postoperative left femur radiograph of the second patient.

**Figure 6:**
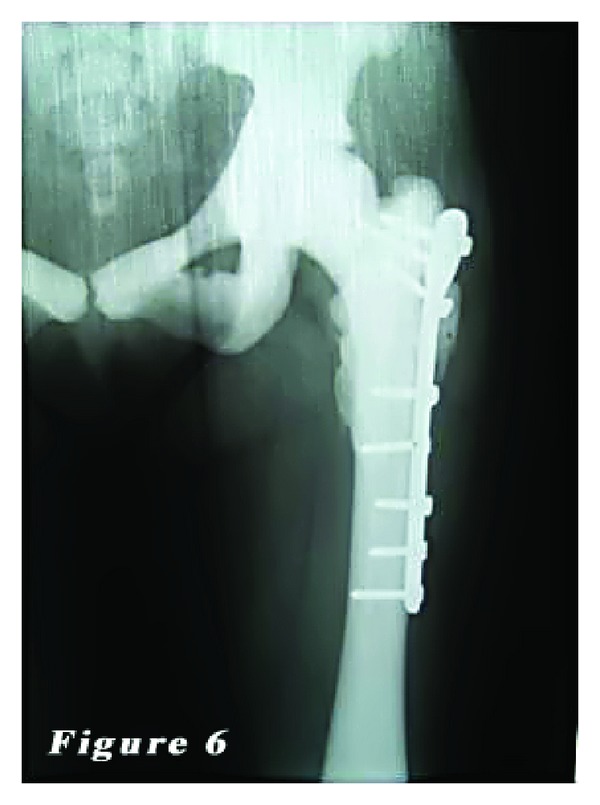
Her last left femur radiography. It looks like fracture union.

**Figure 7:**
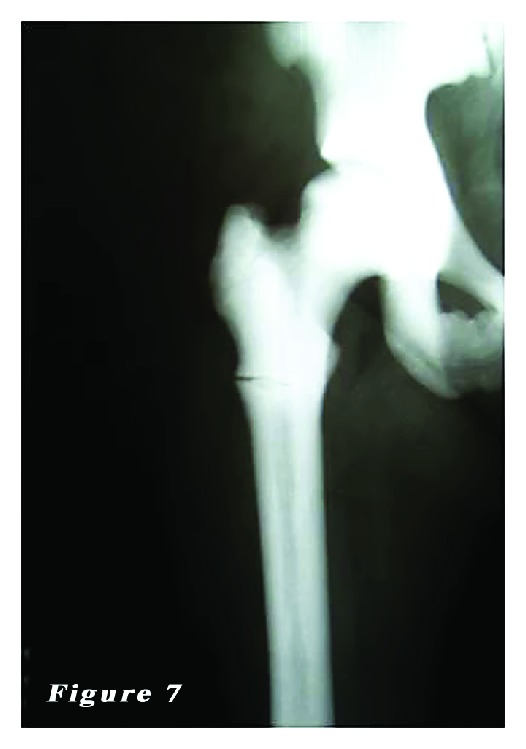
24-year-old female patient (second case) preoperative radiograph of the right femur subtrochanteric stress fractures.

**Figure 8:**
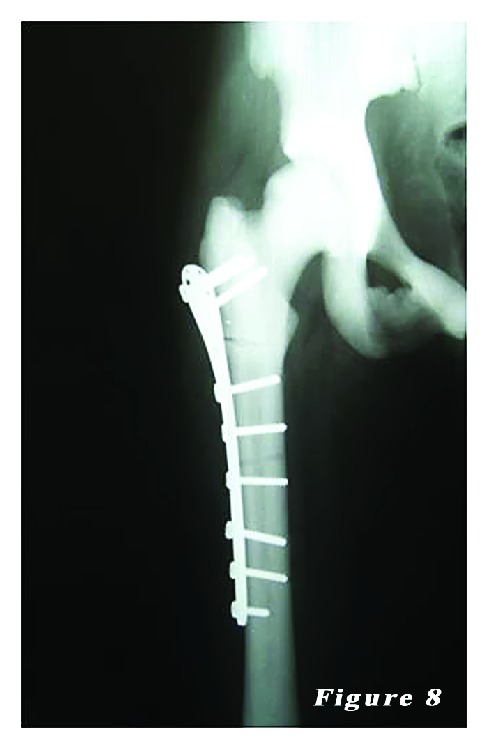
Postoperative right femur radiograph of the second patient.

**Figure 9:**
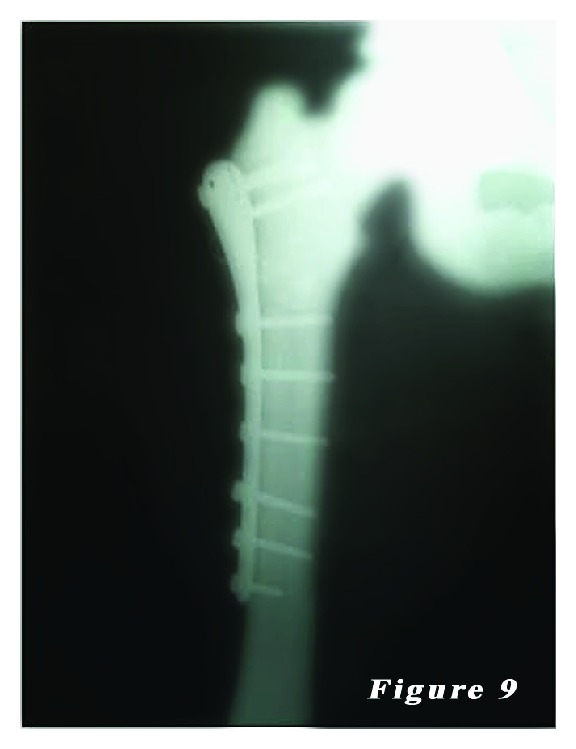
Her last right femur radiography. It looks like fracture union.

*Case *2. ** A 24-year-old female patient was admitted to our emergency department with the complaints of hip pain and the inability to walk. Patient history revealed that her ankle was sprained during a walking fall, after which the patient reported feeling a sharp pain with a clunking sound in the femoral head. Plain X-ray showed an oblique subtrochanteric fracture of the left femur and a fracture line in the lateral cortex of the proximal right femur. The patient was hospitalized with the preliminary diagnosis of osteopetrosis and pathological femoral fracture. Laboratory tests indicated no other pathologies. The family history of the patient did not definitively indicate osteopetrosis. Based on the clinical and radiological findings, the definite diagnosis was osteopetrosis tarda type II along with subtrochanteric osteopetrotic fracture of the left femur and osteopetrotic stress fracture of the right femur. Surgery was scheduled for the treatment of both conditions. Surgery was performed in a supine position under combined anesthesia. Following antiseptic procedures for hips and lower limb, a standard lateral straight incision was performed on the left hip and an open reduction was done. Then, osteosynthesis with internal fixation was performed using three 4.5 mm spongious screws and seven cortical screws and a nine-hole left anatomic plate (Hipokrat, Turkey). Fluoroscopic assessment and intraoperative examination revealed a successful reduction and fixation. Subsequently, a standard lateral straight incision was performed on the right hip and an open reduction was done. Internal fixation was done using nine 4.5 mm cortical screws and a nine-hole right anatomic plate (Hipokrat, Turkey). Drilling was highly difficult; the drill bit was broken twice. No perioperative complication was observed. Bone specimens which were obtained by drilling and the intraoperative biopsy were sent for pathological examination. The patient was followed up in our clinic following surgery. In the postoperative period, there were no scar-related complications. The patient was referred to the rehabilitation program on the third postoperative day, and sutures were removed at 12 days. She was mobilized without loading on the healing fracture of the left side using a crutch at two weeks and with partial loading at six weeks. The histopathological examination confirmed the diagnosis of osteopetrosis. No complications, including infection, nonunion, or avascular necrosis (AVN), were observed during the 12-month follow-up period. The patient was able to walk without pain and using any assistance. Plain radiographs are shown in Figures 4, 5, 6, 7, 8, and 9.

## 3. Discussion

Osteopetrosis, which is a group of conditions, is a heterogeneous hereditary disease characterized by significantly increased bone density due to osteoclast dysfunction. Most patients with infantile or malignant autosomal recessive osteopetrosis die within the first year of life. The life expectancy of patients with intermediate osteopetrosis is moderately reduced, whereas adult patients with benign ADO have a normal life expectancy [10, 13]. Adult benign ADO has two distinct phenotypic variants [1, 5, 8–10, 13]: (i) type I, which is characterized by diffuse sclerosis, predominantly involving long bones, the skull base, and spine, and (ii) type II, which is characterized by radiographic findings of “rugger jersey spine” and “bone within a bone” appearance of the pelvis, in particular. There is no significant difference in radiographic findings of long bones of the appendicular skeleton between these types. Radiographic images contain heterogeneity in both types [1, 5–8, 14, 15]. Serum levels of alkaline phosphatase are reduced in type I and increased in type II. In addition, type I does not present with increased risk of fracture; however, fractures may develop, particularly in long bones, after even minor trauma injuries. Although rare in type I, the incidence of trigeminal neuralgia, facial nerve paralysis, and optic nerve compression is higher in type II. Also, short stature may result from diminished longitudinal growth in patients with type II disease. Other conditions which may be accompanied by ADO type II include hepatosplenomegaly, anemia, renal tubular acidosis, and pancytopenia [14–18].

The half of patients with osteopetrosis is asymptomatic and diagnosed incidentally or based on the presence of a fracture (40%). The disease usually presents without bone marrow involvement. Laboratory values are usually within normal limits. But may be moderate anemia and mild increased serum levels of alkaline phosphatase. Family history or patient history may reveal previous fractures. The most common complaints on admission are bone pain and fractures. In adult benign ADO, bones are prone to fractures due to increased bone density and sclerosis with an increased rate of hip and proximal femoral fractures in type II [4, 5, 10–12, 14–18]. In a study including 42 patients with osteopetrosis tarda, Bénichou et al. [19] reported a fracture rate of 78%. The mean number of fractures was 4.4 and the most common fracture localization was the femur.

In our study, the first case had a positive family history, mild anemia, bilateral loss of vision, diffuse sclerosis, and an osteopetrotic fracture of the right femur. Despite generalized osteosclerosis and osteopetrotic pathological femoral fractures in the second case, her family history was indefinite. She had no neurological deficit and laboratory test results were within normal limits. None of the patients had a previous history of fractures. We diagnosed our patients with ADO type II based on the clinical findings and laboratory and imaging test results.

Several case reports and small-scale case series on the treatment of osteopetrotic fractures are available in the literature [10]. Conservative or surgical modalities are used in the treatment of osteopetrotic fractures, as in the treatment of other fractures. Review of the literature revealed case reports in which conservative treatment modalities were used; however, procedure-related complications including nonunion and coxa vara were also reported [10, 27, 28]. In addition, there are case reports in which various implants (e.g., locking plates, cannulated screws, dynamic condylar screw (DCS), dynamic hip screw (DHS), and intramedullary nailing (IMN)) were used during surgery in the light of methods of osteosynthesis for the surgical treatment of osteopetrotic femoral fractures. Furthermore, case reports regarding the use of hemiarthroplasty and total hip arthroplasty in patients with osteopetrotic fractures can be found in the literature [4–7, 10–12, 17, 23–26].

In a case report and literature review published in 2008, Birmingham and Mchale [10] reported a 56-year-old male case of ADO with an ipsilateral fracture of the left femoral neck along with a subtrochanteric fracture. The patient who was scheduled for surgery refused operation and received conservative treatment. The authors reported that the patient in whom coxa vara and external rotation deformity were observed during the 30-month follow-up period was recovered with a good functional status. Moreover in mentioned article [10] the authors have summarized perfectly the published cases until 2008 with literature review and a table. Therefore we have summarized by comprehensive review of the literature published cases between the years 2008 and 2013 in in Table 1 in this article. And we have tried to discuss this Our cases with current literature.

Osteosynthesis has been the primary method for the surgical treatment of femoral osteopetrotic fractures. In addition, several implantation techniques have been developed thus far [10, 21]. There are case reports in whom IMN was performed in the literature [17, 20]. Kumbaraci et al. [17] presented a 21-year-old female patient with osteopetrosis who underwent open reduction for bilateral subtrochanteric femoral fractures and internal fixation using proximal femoral nail antirotation (PFNA). The patient with a postoperative callus formation was allowed to walk using a crutch at six weeks. Full union was observed bilaterally and the patient was able to walk without using any assistance at 12 months postoperatively. Although proved on X-ray, a medullary canal was absent during surgery. As a result, the fracture line was opened and a proximal line followed by the distal line was operated on using a serial drilling and carving technique to perform PFNA into the created canal. The authors used PFNA for bilateral fractures to achieve early mobilization and to place loading on the healing fractures. In another publication, Cadosch et al. [20] reported a 37-year-old male case of ADO type II with 1/3 proximal shaft fracture of the right femur, which was treated using IMN along with humerus and forearm fractures concomitantly. Contrary to previous studies, several authors reported patients in whom osteosynthesis was performed using various plate-screw systems with the concern of a narrow femoral canal and carving procedure-related possible complications [4–8, 10–12, 21, 23–26]. Although some authors reported excellent outcomes of the plate-screw system, revision surgery due to implant failure was required in several cases [6, 11, 20–28]. There was also an attempt to prevent implant failure through an augmented technique of osteosynthesis with the plate-screw system [7, 25]. Kulkarni et al. [21] reported a 22-year-old male case of ADO type II with the left femoral shaft fracture and a 47-year-old male case of ADO type II with the right subtrochanteric fracture. Both patients who underwent open reduction with internal fixation under combined spinal + epidural anesthesia were successfully treated. In another study, Amit et al. [24] presented 35-year-old and 38-year-old female patients who were successfully treated with a reverse, less invasive stabilization system (LISS) plating due to osteopetrotic subtrochanteric fractures. In addition, Kumar et al. [23] reported a 45-year-old male patient with osteopetrosis in whom the left femoral subtrochanteric fracture was surgically treated. Internal fixation was performed with a DHS instead of IMN due to the presence of a narrow femoral canal. The authors encountered several technical difficulties, including the bending of a drill bit during surgery due to increased bone density and fragile bone structure. They also achieved radiological findings of a good alignment and full union at 11 months postoperatively. They also reported that the patient underwent surgical treatment with DHS due to the subtrochanteric fracture four years earlier. The authors concluded that surgical treatment was more effective to achieve a better functional outcome in the treatment of femoral subtrochanteric fractures under high stress in adults, although conservative treatment was another treatment option.

Furthermore, there are case reports with implant failure in patients with femoral neck fractures undergoing osteosynthesis in the literature. Gandhi et al. [26] reported a 58-year-old male case with a subtrochanteric fracture and neck fracture after a short period on the same side. The patient was treated with plate-screw fixation due to a femoral subtrochanteric fracture. He was also administered DCS due to a femoral neck fracture one year later. However, the plate and DCS were removed and cemented hemiarthroplasty was performed due to the implant failure. The patient was uneventful during the six-month follow-up period. In another study, Ramiah et al. [29] reported a 38-year-old male patient with a femoral neck fracture who underwent internal fixation. Repeated X-ray showed that a number of drills and screws were broken along with the nonunion of the fracture. Total hip arthroplasty was performed in the patient with implant failure. In contrast to these two publications, Bhargava et al. [11] reported a 20-year-old female case of subcapital femoral neck fracture who underwent surgical treatment using a cannulated screw. Complete fracture healing was achieved at six months postoperatively.

In our study, we performed osteosynthesis with a cannulated screw. Complete fracture healing was achieved within the 12-month followup without any complications. Review of the literature revealed no case report in which hemi- or total arthroplasty was performed for osteopetrotic femoral fractures. Despite increased risk for implant failure during the follow-up period, we suggest that osteosynthesis is the primary treatment of choice in the treatment of osteopetrotic femoral fractures. Several surgical-related complications, on the other hand, have been reported in the literature. Several complications such as nonunion, broken plates or screws, recurrent fractures, and infection may be observed [4, 5, 12, 17]. Technical difficulties include bending of drill bits or screws during surgery using drilling or carving due to hard-fragile sclerotic bones and a narrow medullary canal. Slow-speed high-torque electric drills, as well as frequent cooling with physiological saline, clearance of drill grooves, and the use of staggered drill system, have been recommended [12, 17, 25]. However, there is still an increased risk of implant failure and nonunion for internal fixation [12, 17]. To avoid such complications, some authors recommended bone morphogenic protein (BMP) grafting, which stimulates mesenchymal cells and differentiation to osteoblasts thanks to its osteoinductive nature, thereby exerting a positive effect on bone and callus formation and ultimately fracture healing [7, 12]. It should be kept in mind that drilling hard bones and internal fixation may complicate surgery in patients with osteopetrotic fractures. The healing process is also slow in these patients. Orthopedic surgeons should be aware of possible challenges during treatment of such patients. As drilling and carving of the bone as well as insertion of an implant are highly complex procedures, a thorough treatment requires great attention, patience, and effort. The treatment success is based on the appropriate selection of internal fixation and meticulous approach during surgery [12, 30, 31]. On the other hand, implant failure is common with instruments used for drilling and nailing.

## 4. Conclusion

We suggest that surgery is an effective treatment modality in patients with osteopetrotic fractures, although technical difficulties may be experienced and fracture healing is slower than normal. Technical challenges and complications may occur during surgery; however, we believe that osteopetrotic femoral shaft fractures can be successfully treated with plate-screw systems without using any graft, which promotes fracture healing during primary surgery. Furthermore, we recommend internal fixation for the treatment of femoral neck fractures, as it is a relatively biological surgery. Orthopaedics surgeons should be aware of intraoperative technical difficulties and possible postoperative complications during the follow-up period. Investigation would be beneficial for the diagnosis of osteopetrosis such the patient with fractures who has minor trauma history and increased bone density in radiography.

## Figures and Tables

**Table 1:** Osteopetrotic femur fractures were treated surgically: the published cases (2008–2013).

Article	Age(s)	Gender(s)	Femur localization	Surgical treatment	Complication	Follow-up period
Kumbaraci et al. [17]	21 years old	Female	Bilateral subtrochanteric	Intramedullary nail (PFNA)	None	12 months

Cadosch et al. [20]	37 years old	Male	Right proximal	Intramedullary nail	None	6 months

Kulkarni et al. [21]	22 years old	Male	Left shaft	Plate-screw	Unspecified	Unspecified
	47 years old	Male	Right subtrochanteric	Plate-screw	Unspecified	Unspecified

Huang et al. [22]	23 years old	Female	Bilateral shaft	Bilateral plate-screw	None	Unspecified

Kumar et al. [23]	45 years old	Male	Bilateral subtrochanteric	Dynamic Hip Screw	None	11 months

Golden and Rodriguez [7]	27 years old	Male	Bilateral subtrochanteric	Dynamic Condylar Screw	None	3 years

Amit et al. [24]	35 years old	Female	Right subtrochanteric	Locking plate	Contralateral stress fracture	23 weeks
	38 years old	Female	Left subtrochanteric	Locking plate	None	21 weeks

Sen et al. [25]	Mean 26	4 male/1 female	4 subtrochanteric (one of them bilateral)	Locking plate	None	3 months

Bhargava et al. [11]	48 years old	Female	Bilateral shaft	Locking plate	Bilateral delayed union	3 years

Gandhi et al. [26]	58 years old	Male	Right neck	Hemiarthroplasty	None	6 months

Sonohata et al. [6]	61 years old	Female	Right subtrochanteric	Hemiarthroplasty	None	2 years
